# Delays in Education and Leadership for Youth with Disabilities: Lessons from the TamTam Project

**DOI:** 10.4102/ajod.v13i0.1323

**Published:** 2024-01-24

**Authors:** Nfotoh P. Kesah, Wanka’a R. Che, Agbor A. Agbor Junior, Lynn Cockburn

**Affiliations:** 1 Department of Communication and Advocacy, Hope Social Union for the Visually Impaired, Bamenda, Cameroon; 2 Department of Conflict Resolution, Faculty of Laws and Political Sciences, University of Buea, Cameroon; 3 Department of Sociology and Anthropology, Faculty of Social and Management Sciences, University of Buea, Cameroon; 4 Department of Medical Sociology, Anthropology and Psychology, Faculty of Health Science, Experiential Higher Institute of Science and Technology, Yaounde, Cameroon; 5 School of Rehabilitation Science, Faculty of Science, McMaster University, Hamilton, Ontario, Canada

## Introduction

Ensuring access to education for students with disabilities requires collective efforts from governments, teachers, parents, and students themselves (UNESCO 2019). There are variations in educational trajectories for people with disabilities in Africa, and students with disabilities in African settings are often denied education or have their education delayed because of many factors (Engelbrecht et al. 2016; Okyere et al. 2019; UNESCO 2019). For example, despite active governmental efforts in Tanzania, difficulties still exist between rhetoric and reality (Adugna et al. 2022; Braun 2020), and many students with disabilities are either not in school or have less than optimal experiences.

In low-income African nations, young people with disabilities can be confronted with several potential problems in adapting to their impairments (UN Department of Economic and Social Affairs 2019). Emotional adjustment can be related to living with impairments, family supports, experiences of discrimination, adopting a ‘disability’ identity, and the socioeconomic ramifications of becoming people with disabilities. In addition to these factors, dropout rates from university level education are affected by substandard housing environments, community opinions that persons with disabilities do not require education, terrain that is difficult to navigate, and cultural beliefs ascribing disability to wrongdoing. Individual experiences and responses are likewise distinct, reflecting the multifaceted nature of each person’s situation. Researchers in African universities are beginning to investigate these disparities (McKinney & Swartz 2014; Molteno et al. 2002).

This article stems from work in Cameroon, where laws and frameworks for inclusive education have been introduced with very slow follow up and implementation (Cockburn et al. 2017; Ndjouma 2020). The right to education has not been realised for persons with disabilities in Cameroon, with approximately one in every 10 children with disabilities having a chance to attend school and few schools having adequate systems in place (Tah & Mufuh 2018). Despite some government actions taken to increase access and inclusion in some government schools, it has been estimated that 9 of 10 children with disabilities in the country did not attend school (Tah & Mufuh 2018). Although improvement in policies, teacher training, and increase in enrolment of students with disabilities are beginning to show (Cockburn et al. 2017; Mbibeh 2013; Opoku, Mprah & Saka 2016; Tcheimegni 2018), it is clear that many Cameroonian children with disabilities still either start school late or never have access at all (SightSavers 2022; Wodon et al. 2018).

The Prime Ministerial decree of 2018 stated that Law N° 2010/002 on the protection and promotion of persons with disabilities should be put into effect. Articles 4 and 5 of the 2010 law call for children with disabilities to have equal access to mainstream schooling and vocational training programmes, and that teachers are expected to maintain high standards, including learning sign language and Braille. The government of Cameroon recently announced a new policy which recognised that youths with disabilities face barriers in attending school, and subsequently applying for government positions. Therefore, they now permit persons with disability cards an additional 5 years – from 30 years to 35 years – to apply for competitive entrance to civil service, recognising that it can take them longer to complete school (Government of Cameroon, 19 May 2022).

## The TamTam project of the Hope Social Union for the Visually Impaired

The Hope Social Union for the Visually Impaired (HSUVI) is a Cameroonian, non-governmental association of persons with visual impairment. Hope Social Union for the Visually Impaired focuses especially on areas such as employment, policy formulation, and general human rights. In 2022, HSUVI was funded for a project to develop leadership attitudes and skills in youth with disabilities from all parts of Cameroon, called TamTam Leadership Project. The goal was to develop leadership abilities in youth with disabilities using a ‘train the trainers’ model. Specific skills included advocacy, how to mainstream disability in sectors such as media and education, how to develop inclusive businesses, and engage in community development through effective participation and representation. The first group of trainers were 22 university students with disabilities aged 18 to 25, from all 10 regions of the country, who participated in a 2-day in-person training workshop, with before and after support and resource material. They then returned to their home regions to train others. The objective of this model of training was to have a meaningful impact for the youth with disabilities themselves, many of whom had limited life experiences, so as to model the changes that were desired. When these youth leaders returned to their home regions, they hosted similar workshops for other youth with disabilities.

As we implemented the project, the initial goal of having youth in this age range did not appear to be achievable. During the recruitment process, the organising team observed that many of the youth applying or being proposed were older than the targeted age of 25 years. The planning team began to explore the situation and had many discussions about it. In doing so, we realised that there is little published work to address the issue of age in disability experiences in Cameroon, and in other parts of Africa. We also recognised that there are gaps in the literature explaining why youth with disabilities are delayed in their education in Cameroon (Cockburn et al. 2017; ICED Report 2014). This article is a result of these discussions, including reflections on the new policy, and was part of the programme evaluation process for the project.

Therefore, the purpose of this article is to explore the situation and the evidence related to why youth with disabilities are older when entering and going through the education system in Cameroon. We anticipate that although we focus on Cameroon, the findings will have implications for other African settings. The goal is to assist non-governmental organisations to consider effective strategies for the inclusion of people with disabilities in youth-oriented programmes.

## Methods

Once the general need for more understanding on the topic of age delays for students with disabilities was identified, we expanded our search for relevant information. The information for this article comes from a review of documents, including legislation, scholarly articles and popular media (e.g. blogs, newspapers), and the discussions with the TamTam Leadership group. The TamTam Leadership incorporated a detailed and extensive programme evaluation during the 1 year of its operation (2022). As it was part of the programme evaluation process of the TamTam, and we are not using any specific information from participants, research ethics was not required. Information about participants and their evaluative feedback were collected through Google forms at point of application, acceptance, and after the training workshops. These forms collected demographic and other information about participants including age, current organisational affiliations, personal goals, and leadership experiences. We found that there were many challenges, which prevented university students from applying and participating, which we have divided into four themes.

### Ethical considerations

We did not apply for a waiver for ethical consideration because it was not a research study. The primary purpose of the project was to provide a programme. This article is based on some work that occurred to assess the functioning of that programme in order to improve it going forward. Publication was a secondary goal.

## Results and reflections from gathering the applications: What happened?

*Theme 1: In all of the regions, there were many barriers resulting in relatively few applicants*.

The TamTam Project targeted universities because they have established associations of students with disabilities, and we estimated that many of these students would be interested in leadership training opportunities. We are aware of the barriers facing youth with disabilities to even get to higher education, but we decided to focus on university because of the potential for these students to have the skills to replicate the workshops in their own communities.

It is resource intensive to attend school (e.g. time, money, personal energy) and students have little extra resource for other activities. Several students (or their parents) did not have the confidence that they could travel out of a familiar area. Some students did not have the necessary mobility devices such as wheelchairs or tricycles, and so could not travel long distances to participate. Some have to be carried or have additional medical needs and costs, reducing their interest in leaving their home area where they have trusted care providers. The TamTam Project tried to address some of these concerns by providing individualised support to participants through phone calls and WhatsApp messages and covering the cost of transportation.

Many talked about the extra costs for transportation (e.g. a taxi might charge double to carry a person with a disability), and even though these costs would have been covered by the programme, they were reluctant to take the chance of getting stranded along the way. Some students reported that they did not apply because they could not access the application form because of a lack of devices or skills to complete the application, and they did not have anyone to support them to do so. Finally, there may be other reasons that we are not aware of, which prevented students from applying.

*Theme 2: Many people disregarded the age criteria and applied even if they were over the age*.

Many of the applicants for the centralised training were above the age range, including several who were over 30. The organisers were then faced with the dilemma of what to do – reject them outright or adjust the acceptable age range. In addition, a small number did not provide an age, and we had to follow up with them. In doing so, we found that some applicants were angry with the programme that an age limit was imposed, and stated that they wanted to apply even though they were over 25. From talking with these applicants and others, we learned that they felt they did not have opportunities when they were younger (they were not previously included in such workshops), and they now wanted to participate. The number of applicants from the over 25 group showed that there is a significant need for this kind of training and engagement among older students and community members. We think this interest also reflects the shortage of work and training opportunities for people with disabilities.

*Theme 3: The leaders in the groups know only a portion of youth with disabilities in their universities and communities*.

Each region was set up to recruit youth, mostly through partnerships with student organisations in the university systems. We know from anecdotal reports that many students with disabilities are not members of associations, and might try to minimise or hide their disability because of stigma and discrimination. In addition, perhaps the communication between and within the associations was not convincing enough to motivate student members to submit their applications for the training. Universities also often do not have good records of students with disabilities.

*Theme 4: A lack of a culture of youth leadership and the possibility for youth with disabilities to see themselves as leaders*.

Anecdotal reports were that many youth with disabilities either directly said they are not interested in leadership training opportunities because they do not see how it will benefit them personally (e.g. they do not see it leading to employment), or the community leaders in some areas did not fully believe that students could be leaders. For example, Regional Committee members in the Littoral Region said that very few people applied for the training because they did not think it would benefit them enough. In this region, there did appear to be more interest with older youth, perhaps because they had more experience and could see the broader benefits.

It was also suggested that the general lack of self-esteem that many youth with disabilities have could impact their willingness to participate – if youth do not see themselves as even being potential leaders, they would not try. Youth might also see themselves as people to be taken care of, not as leaders, and feared that if they participated, they might lose some of the current support received from others.

Putting it all together: What does this experience show?

Youth with disabilities continue to be marginalised, in the broader community, in university settings, and even in the disability community. The experience from this project suggests that it does take longer for some people to accept opportunities, to go through school, and to become leaders. We have learned to think carefully about putting age ranges for programmes for ‘youth’ with disabilities. In the end, we had several participants in the 26 to 30 age range, and a few who were even older.

Depending on their goals, some programmes might want to consider having ‘younger youth’ and ‘older youth’ categories. While inclusion of persons with disabilities by age consideration may be sufficient for the protection and mainstreaming of disability in some programmes, we believe that other programmes may require more than just the prerequisite of age consideration to provide equity in access and opportunities for leadership and participation in community actions to youth with disabilities in Cameroon. Thus, our experiences suggest that programmes need to carefully think about and develop a rationale for age criteria when setting up programmes.

## References

[CIT0001] Adugna, M., Ghahari, S., Merkley, S. & Rentz, K., 2022, ‘Children with disabilities in Eastern Africa face significant barriers to access education: A scoping review’, *International Journal of Inclusive Education* 26, 1–17. 10.1080/13603116.2022.2092656

[CIT0002] Braun, A.M.B., 2022, ‘Barriers to inclusive education in Tanzania’s policy environment: National policy actors’ perspectives’, *Compare: A Journal of Comparative and International Education* 52(1), 110–128. 10.1080/03057925.2020.1745057

[CIT0003] Cockburn, L., Hashemi, G., Noumi, C., Ritchie, A. & Skead, K., 2017, ‘Realizing the educational rights of children with disabilities: An overview of inclusive education in Cameroon’, *Journal of Education and Practice* 8(6), 1–11, viewed 10 December 2023, from https://eric.ed.gov/?id=EJ1133086.

[CIT0004] Engelbrecht, P., Nel, M., Smit, S. & Van Deventer, M., 2016, ‘The idealism of education policies and the realities in schools: The implementation of inclusive education in South Africa’, *International Journal of Inclusive Education* 20(5), 520–535. 10.1080/13603116.2015.1095250

[CIT0005] International Centre for Evidence in Disability (ICED), 2014, *The North West Cameroon disability study country report*, London School of Hygiene and Tropical Medicine (LSHTM), viewed 14 August 2022, from https://www.lshtm.ac.uk/sites/default/files/2019-06/Cameroon-Country-Report.pdf.

[CIT0006] Mbibeh, L., 2013, ‘Implementing inclusive education in Cameroon: Evidence from the Cameroon Baptist Convention Health Board’, *International Journal of Education* 5(1), 52–68. 10.5296/ije.v5i1.3279

[CIT0007] McKinney, E.L. & Swartz, L., 2022, ‘Integration into higher education: Experiences of disabled students in South Africa’, *Studies in Higher Education* 47(2), 367–377. 10.1080/03075079.2020.1750581

[CIT0008] Molteno, G., Molteno, C.G., Finchilescu, G. & Andrew, D., 2002, ‘Behavioural and emotional problems in children with intellectual disability attending special schools in Cape Town, South Africa’, *Journal of Intellectual Disability Research* 45(Pt 6), 515–520. 10.1046/j.1365-2788.2001.00368.x 11737538

[CIT0009] Moriña, A. & Biagiotti, G., 2021, ‘Academic success factors in university students with disabilities: A systematic review’, *European Journal of Special Needs Education* 37(5), 729–746. 10.1080/08856257.2021.194000

[CIT0010] Ndjouma, M., 2020, ‘Inclusive education: The forms of violation of childrens? Rights and school dropouts in the Kadey Division: East Region of Cameroon’, *International Journal of Scientific Research and Management (IJSRM)* 8(4), 1–6. 10.18535/ijsrm/v8i04.el01

[CIT0011] Okyere, C., Aldersey, H.M., Lysaght, R. & Sulaiman, S.K., 2019, ‘Implementation of inclusive education for children with intellectual and developmental disabilities in African countries: A scoping review’, *Disability and Rehabilitation* 41(21), 2578–2595. 10.1080/09638288.2018.1465132 29693469

[CIT0012] Opoku, M.P., Mprah, W.K. & Saka, B.N., 2016, ‘Participation of persons with disabilities in political activities in Cameroon’, *Disability and the Global South* 3(2), 980–999, viewed 10 December 2023, from https://hdl.handle.net/102.100.100/567706.

[CIT0013] Peterson, A., Hattam, R., Zembylas, M. & Arthur, J. (eds.), 2016, *The Palgrave International Handbook of Education for Citizenship and Social Justice*. Palgrave Macmillan, UK. 10.1057/978-1-137-51507-0

[CIT0014] SightSavers, 2022, *Investigating the cost and budget impact of inclusive education in Cameroon*, viewed 10 December 2023, from https://research.sightsavers.org/project/investigating-the-cost-and-budget-impact-of-inclusive-education-in-cameroon/.

[CIT0015] Tah, P. & Mufuh, R., 2018, *CBC health services champions access to education for children with disabilities*, viewed 10 December 2023, from https://cbchealthservices.org/cbchs-champions-access-to-education-for-children-with-disabilities/.

[CIT0016] Tcheimegni, E., 2018, ‘Including students with special needs in a mainstream classroom in Cameroon’, Master’s thesis, viewed 10 December 2023, from https://scholars.fhsu.edu/theses/593.

[CIT0017] UN Department of Economic and Social Affairs, 2019, *Disability and development report realizing the sustainable development goals by, for and with persons with disabilities*, p. 365, United Nations.

[CIT0018] UNESCO, 2019, *The right to education for persons with disabilities*, UNESCO Digital Library, viewed 10 December 2023, from https://unesdoc.unesco.org/ark:/48223/pf0000371249.

[CIT0019] Wodon, Q., Male, C., Montenegro, C. & Nayihouba, A., 2018, ‘The challenge of inclusive education in sub-Saharan Africa’, in *The price of exclusion: Disability and education*, World Bank, Washington, DC, viewed 10 December 2023, from https://openknowledge.worldbank.org/handle/10986/31005.

